# Supplementary material for: The interactome of intact mitochondria by cross-linking mass spectrometry provides evidence for coexisting respiratory supercomplexes
Source: Mol Cell Proteomics. 2017 Dec 8;17(2):216–32. doi: 10.1074/mcp.RA117.000470 (PMC5795388; doi:10.1074/mcp.RA117.000470)
Supplement: Supplemental Data [file supp_RA117.000470_133922_0_supp_23978_fzffwf.zip › spectra_annotation/mito_DR_spectra_annotation/383-1-10-1-6-1.pdf]

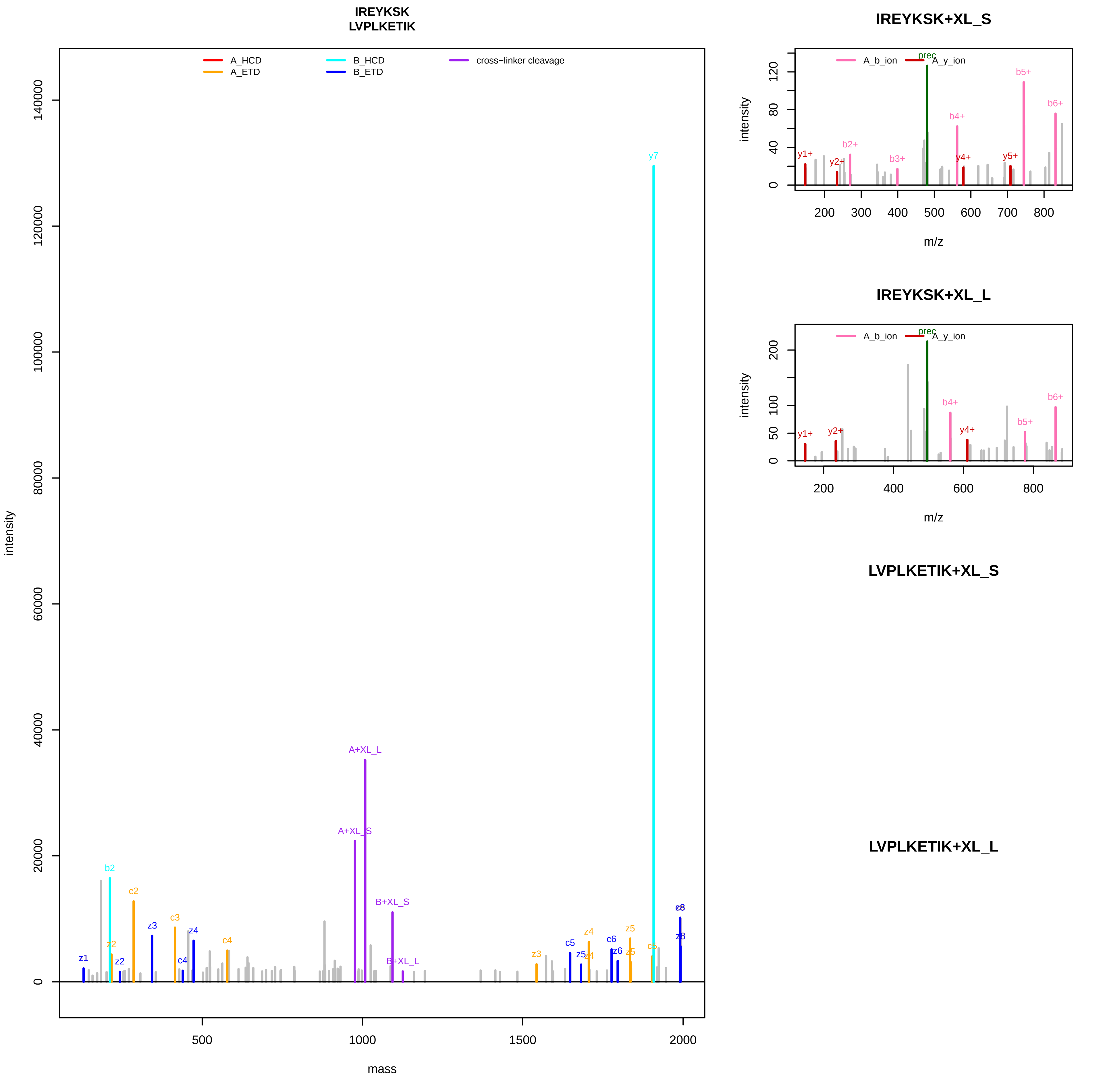

IREYKSK
LVPLKETIK
A_HCD
A_ETD
B_HCD
B_ETD
cross−linker cleavage
intensity
mass
y7
A+XL_L
A+XL_S
B+XL_S
B+XL_L
b2
c2
c3
c4
c5
c6
z1
z2
z3
z4
z5
z6
z8
IREYKSK+XL_S
A_b_ion
A_y_ion
prec
y1+
y2+
y4+
y5+
b2+
b3+
b4+
b5+
b6+
m/z
IREYKSK+XL_L
LVPLKETIK+XL_S
LVPLKETIK+XL_L
